# Supplementary material for: LQFM289: Electrochemical and Computational Studies of a New Trimetozine Analogue for Anxiety Treatment
Source: Int J Mol Sci. 2023 Sep 26;24(19):14575. doi: 10.3390/ijms241914575 (PMC10572256; doi:10.3390/ijms241914575)
Supplement: Supplementary file 1 [file ijms-24-14575-s001.zip › ijms-2577559-supplementary.pdf]

Supplementary Materials

Table S1. Crystal data and structure refinement for LQFM289 (**3**).

Identification code	ORSY23024	
Empirical formula	C19 H29 N O3	
Formula weight	319.43	
Temperature	287(2) K	
Wavelength	0.71073 Å	
Crystal system	Monoclinic	
Space group	P 21/n	
Unit cell dimensions	a = 17.3332(14) Å	α= 90°.
	b = 12.2252(9) Å	β= 110.427(3)°.
	c = 18.9650(15) Å	γ = 90°.
Volume	3766.0(5) $Å^3$	
Z	8	
Density (calculated)	1.127 Mg/m^3	
Absorption coefficient	0.075 mm^{-1}	
F(000)	1392	
Crystal size	0.347 x 0.237 x 0.060 mm^3	
Theta range for data collection	1.372 to 25.029°.	
Index ranges	-20<=h<=20, -14<=k<=14, -22<=l<=22	
Reflections collected	75266	
Independent reflections	6655 [R(int) = 0.1073]	
Completeness to theta = 25.000°	100.0 %	
Refinement method	Full-matrix least-squares on F^2	
Data / restraints / parameters	6655 / 0 / 430	
Goodness-of-fit on F^2	1.130	
Final R indices [I>2sigma(I)]	R1 = 0.1788, wR2 = 0.4337	
R indices (all data)	R1 = 0.2087, wR2 = 0.4521	
Extinction coefficient	0.0041(15)	
Largest diff. peak and hole	0.880 and -0.449 e.$Å^{-3}$	

Table S2. Crystal data and structure refinement for TRIMETOZINE (**2**).

Empirical formula	C14 H19 N O5	
Formula weight	281.30	
Temperature	297(2) K	
Wavelength	0.71073 Å	
Crystal system	Triclinic	
Space group	P -1	
Unit cell dimensions	a = 8.6525(4) Å	α= 64.2340(10)°.
	b = 9.5424(4) Å	β= 77.059(2)°.
	c = 9.8544(4) Å	γ = 71.725(2)°.
Volume	692.12(5) Å^3	
Z	2	
Density (calculated)	1.350 Mg/m^3	
Absorption coefficient	0.103 mm^{-1}	
F(000)	300	
Crystal size	0.402 x 0.272 x 0.168 mm^3	
Theta range for data collection	2.307 to 29.700°.	
Index ranges	-12<=h<=12, -13<=k<=13, -13<=l<=13	
Reflections collected	38484	
Independent reflections	3918 [R(int) = 0.0463]	
Completeness to theta = 25.000°	100.0 %	
Refinement method	Full-matrix least-squares on F^2	
Data / restraints / parameters	3918 / 0 / 184	
Goodness-of-fit on F^2	1.084	
Final R indices [I>2sigma(I)]	R1 = 0.0497, wR2 = 0.1466	
R indices (all data)	R1 = 0.0618, wR2 = 0.1529	
Largest diff. peak and hole	0.359 and -0.226 e.Å^{-3}	

Table S3. *Selected bond lengths (Å)*

	LQFM289-A	LQFM289-B	TMZ	TUJTAV	OPT
O1A—C1A	1.226 (9)	1.238 (10)	1.2240(15)	1.223(3)	1.2157
O2A—C5A	1.373 (8)	1.333 (9)	1.3753(13)	1.375(1)	1.3633
O3A—C9A	1.405 (12)	1.378 (14)	1.4180(19)	1.414(3)	1.4137
O3A—C11A	1.414 (12)	1.383 (13)	1.4210(17)	1.419(2)	1.4098
N1A—C1A	1.360 (10)	1.357 (11)	1.3544(16)	1.354(2)	1.3571
N1A—C8A	1.455 (10)	1.438 (11)	1.4634(15)	1.463(2)	1.4555
N1A—C10A	1.447 (10)	1.441 (10)	1.4623(16)	1.462(3)	1.4571
C(1A)-C(2A)	1.482(10)	1.487(10)	1.5012(15)	1.500(2)	1.4956

Table S4. *Selected bond angles (°)*

	LQFM289-A	LQFM289-B	TMZ	TUJTAV	OPT
C(1A)-N(1A)-C(8A)	120.4(7)	120.5(8)	120.21(11)	120.3(1)	117.99
C(1A)-N(1A)-C(10A)	124.7(6)	125.0(6)	125.18(10)	125.2(1)	124.46
C(10A)-N(1A)-C(8A)	112.1(6)	114.5(8)	112.77(10)	112.8(1)	112.14
C(9A)-O(3A)-C(11A)	111.3(7)	110.2(8)	109.65(10)	109.6(1)	111.42
O(1A)-C(1A)-N(1A)	120.1(6)	120.1(7)	122.17(11)	122.1(1)	121.67
O(1A)-C(1A)-C(2A)	119.7(6)	120.4(7)	120.18(10)	120.2(1)	120.70
N(1A)-C(1A)-C(2A)	120.1(6)	119.4(7)	117.43(10)	117.5(1)	117.55

Table S5. *Selected torsion angles (°)*

	LQFM289-A	LQFM289-B	TMZ	TUJTAV	289A-OPT
O1-C1-C2-C7	36(1)	50(1)	46.6(2)	46.4(2)	37.61
O1-C1-N1-C8	6(1)	8(1)	5.4(2)	5.8(2)	2.91
O1-C1-N1-C10	-154.0(8)	-170.7(9)	-158.1(1)	-158.1(1)	-149.03
C2-C1-N1-C10	30(1)	13(1)	27.3(2)	26.8(2)	34.15
C1-N1-C8-C10	-162(1)	-179(1)	-165.3(2)	-165.7(2)	-155.25
N1-C8-C9-O3	55(1)	-56(1)	-56.7(2)	-55.7 (2)	55.8

Table S6. *Hydrogen bonds for LQFM289 [Å and °]*

D-H...A	d(D-H)	d(H...A)	d(D...A)	<(DHA)
O(2B)-H(2B)...O(1B)#1	0.82	2.21	2.819(9)	131.0
O(2A)-H(2A)...O(1A)#2	0.82	2.20	2.768(7)	127.0

Symmetry transformations used to generate equivalent atoms:

#1 -x+3/2,y+1/2,-z+1/2 #2 -x+1/2,y+1/2,-z+1/2

Table S7. *Hydrogen bonds for TMZ [Å and °].*

D-H...A	d(D-H)	d(H...A)	d(D...A)	<(DHA)
C(13)-H(13A)...O(5)	0.96	2.51	3.0495(18)	115.6

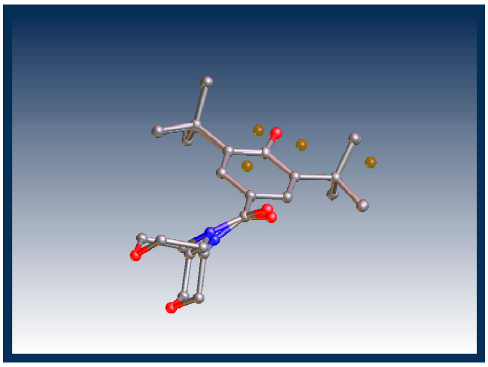

Figure S1. Superposition of LQFM289A and LQFM 289B by a proper transformation matrix.

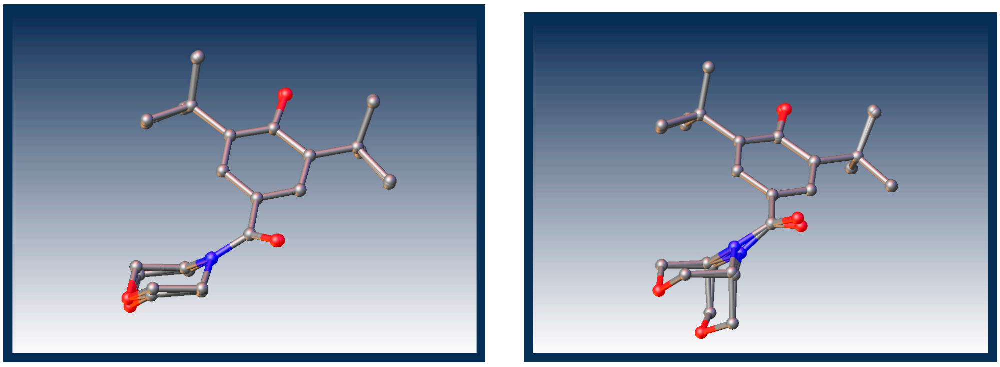

(**a**) (**b**)

Figure S2. Superposition of: (**a**) LQFM289A and LQFM289A-OPT and (**b**) LQFM289B and LQFM289A-OPT.

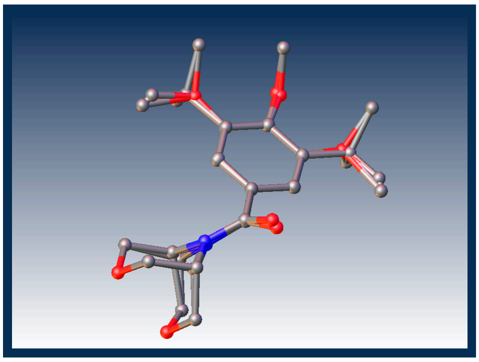

(**a**)

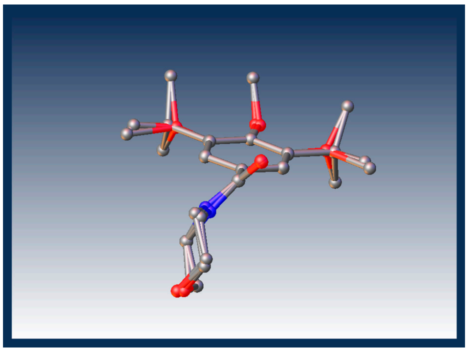

(**b**)

Figure S3: Superposition of: (**a**) LQFM289A and TMZ and (**b**) LQFM289B and TMZ. In both, TMZ was superposed to LQFM289A by an improper matrix transformation.

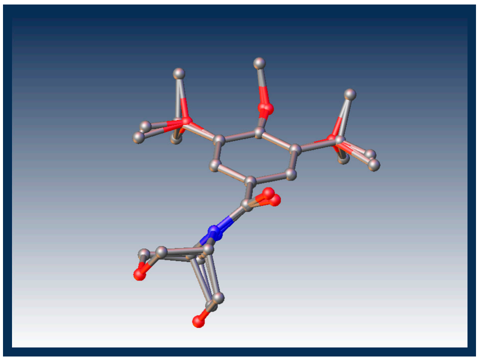

Figure S4. Superposition of LQFM289A and TUJTAV by an improper transformation matrix.
